# Results from a long-term open-label extension study of adjunctive buprenorphine/samidorphan combination in patients with major depressive disorder

**DOI:** 10.1038/s41386-019-0451-3

**Published:** 2019-06-29

**Authors:** Michael E. Thase, Arielle D. Stanford, Asli Memisoglu, William Martin, Amy Claxton, J. Alexander Bodkin, Madhukar H. Trivedi, Maurizio Fava, Miao Yu, Sanjeev Pathak

**Affiliations:** 1 Department of Psychiatry, Perelman School of Medicine, University of Pennsylvania School of Medicine, 3535 Market Street, Suite 670, Philadelphia, PA 19104-3309 USA; 2 Alkermes, Inc., Waltham, MA USA; 3 McLean Hospital, Belmont, MA USA; 4 Harvard Medical School, Boston, MA USA; 5 University of Texas Southwestern Medical Center, Dallas, TX USA; 6 Massachusetts General Hospital, Boston, MA USA

**Keywords:** Drug development, Outcomes research

## Abstract

Buprenorphine/samidorphan (BUP/SAM; ALKS 5461) is an investigational opioid system modulator for the adjunctive treatment of patients with major depressive disorder (MDD), who did not respond adequately to prior antidepressant therapy (ADT). FORWARD-2, an open-label extension study, assessed long-term safety and tolerability of adjunctive BUP/SAM treatment in these patients. Patients from four short-term trials and de novo patients were enrolled; all had confirmed MDD and a current major depressive episode lasting 2–24 months. Patients were treated with an established ADT for ≥8 weeks before receiving sublingual, adjunctive BUP/SAM 2 mg/2 mg for up to 52 weeks. Safety (primary objective) was assessed via adverse events (AEs), the Columbia-Suicide Severity Rating Scale, and the Clinical Opiate Withdrawal Scale (COWS). Exploratory evaluation of efficacy was done using the Montgomery–Åsberg Depression Rating Scale (MADRS). Of 1485 patients, 50% completed the study and 11% discontinued due to AEs. AEs of nausea, headache, constipation, dizziness, and somnolence, each occurred in ≥10% of patients. There was no evidence of increased suicidal ideation or behavior. Euphoria-related AEs were uncommon (1.2%). Following abrupt BUP/SAM discontinuation, “drug withdrawal” AEs were infrequent (0.4%), and the incidence of COWS categorical worsening after abrupt drug discontinuation was low (6.5%). Improvements in mean MADRS scores were maintained until study end, suggesting durability of antidepressant effect in patients continuing treatment. BUP/SAM was generally well tolerated, with a low risk of abuse and an AE profile consistent with those seen in placebo-controlled studies. Withdrawal reports were uncommon and of limited clinical impact.

## Introduction

Major depressive disorder (MDD) is a leading cause of global disability [1] and morbidity [2] and is among the most common mental health disorders [3]. Nearly two-thirds of patients receiving first-line drugs for depression fail to achieve remission, more than half fail to respond [4], and there is substantial risk of relapse among patients who do achieve full remission [5]. Switching is recommended if the initial treatment is unsuccessful but is often only modestly effective [4]. Adjunctive therapy is usually the next step if switching is unsuccessful [6, 7].

Many pharmacotherapies that are currently approved for treatment of MDD, including selective serotonin reuptake inhibitors (SSRIs), serotonin–norepinephrine reuptake inhibitors (SNRIs), and bupropion, target monoaminergic pathways [8, 9]. The only approved adjunctive therapies for MDD are atypical drugs for psychosis. These drugs also work through monoamine modulation and are associated with significant and sometimes permanent side effects, including metabolic abnormalities, weight gain, and movement disorders (such as akathisia and tardive dyskinesia) [10, 11].

There is an urgent need for novel drugs for depression and adjunctive agents with nonmonoaminergic mechanisms of action. The development of new medications should include approaches to minimizing adverse effects as well as discontinuation syndromes [12].

The endogenous opioid system plays a critical role in fundamental psychological processes affected by depression (e.g., motivation, social functioning/attachment) [13–15], and evidence supports opioid modulation as a potential treatment target for MDD [15–17]. Buprenorphine (BUP), a μ-opioid receptor partial agonist and κ-opioid receptor antagonist, has shown potential antidepressant activity in open-label [18–21] and double-blind, placebo-controlled studies in patients with MDD [22–25]. However, the use of opioids in MDD treatment has been limited by their risk of abuse and dependence [22]. Samidorphan (SAM), a µ-opioid receptor antagonist with a low intrinsic activity at κ- and δ-opioid receptors, was combined with BUP to mitigate its abuse and dependence potential while preserving its antidepressant effects [26, 27].

Four randomized, double-blind, placebo-controlled trials were conducted to evaluate efficacy, safety, and tolerability of buprenorphine/samidorphan (BUP/SAM; ALKS 5461) in patients who experienced an inadequate response to one or two drugs for depression during their current major depressive episode (MDE) [24, 27, 28]. To assess the long-term effects of BUP/SAM, eligible patients from these acute treatment studies (including those who completed and those who were ineligible for the double-blind treatment period), as well as de novo patients, were enrolled in FORWARD-2 (*FORWARD:* Focused on Results with a Rethinking of Depression), a 52-week open-label study.

## Patients and methods

### Study design and patients

FORWARD-2 (ClinicalTrials.gov ID: NCT02141399) was an open-label, 52-week study to evaluate the long-term safety and tolerability of BUP/SAM 2 mg/2 mg as adjunctive therapy to drugs for depression for the treatment of MDD. FORWARD-2 enrolled patients from 14 May, 2014 to 31 October, 2017, and was conducted in the United States (138 sites), Bulgaria (9), Germany (9), Canada (5), Hungary (4), Australia (4), and Poland (4).

Patients who completed the FORWARD-1 (NCT02085135; 8-week trial), FORWARD-3 (NCT02158546; 10-week trial), FORWARD-4 (NCT02158533; 12-week trial), or FORWARD-5 (NCT02218008; 11-week trial) studies within 10 days of FORWARD-2 entry were eligible. Three of the aforementioned studies included a prospective lead-in period to identify patients for the blinded randomized phase. Patients who responded during this prospective lead-in but did not meet the criterion of remission (Montgomery–Åsberg Depression Rating Scale [MADRS] ≤10) were eligible for FORWARD-2. Patients who did not respond during the prospective lead-in and were not in remission were eligible to enter the blinded randomized phase of FORWARD-3, FORWARD-4, or FORWARD-5. Patients who completed participation in these studies could enroll in FORWARD-2. De novo patients who had not participated in a prior study of BUP/SAM within the last 2 years were also enrolled. Patients who participated in FORWARD-1, FORWARD-3, FORWARD-4, or FORWARD-5 who did not complete their respective study (i.e., withdrew for any reason) were not eligible for FORWARD-2.

All eligible patients met *Diagnostic and Statistical Manual for Mental Disorders*, 4th Edition, Text Revision (DSM-IV-TR) criteria for MDD, were experiencing an MDE lasting 2–24 months and were aged 18–70 years. Patients who had completed a prior BUP/SAM study and de novo patients were required to have demonstrated one or two inadequate responses to a drug for depression (verified by historical records or by prospectively collected response data). Inadequate response was defined as <50% reduction in depressive symptom severity to an adequate dose of a drug for depression for ≥8 weeks (inclusive of up to 3 weeks for titration into the adequate dose range) during the current MDE. For de novo patients, response to a drug for depression was assessed by the Massachusetts General Hospital Antidepressant Treatment Response Questionnaire. For patients who participated in a prior BUP/SAM study, response was not reassessed.

All patients continued their current drug for depression throughout the study; the dosage could be adjusted based on tolerability, but no change in therapy was allowed. Patients received a once-daily sublingual tablet of BUP/SAM 2 mg/2 mg as adjunctive treatment for up to 52 weeks. Patients newly initiating BUP/SAM 2 mg/2 mg (those who received placebo or no study drug prior to FORWARD-2 entry) and patients who had received a lower dose or paused BUP/SAM treatment during the follow-up period in the previous study underwent a 1-week titration period during week 1. Titration was blinded for patients continuing from the randomized controlled trials (FORWARD-3 and FORWARD-5) to maintain the blindness of randomized treatment in the prior study and unblinded for patients from FORWARD-4, as they were off study drug prior to entry. At study completion, patients stopped BUP/SAM treatment without a taper.

The study protocol was reviewed by an independent ethics committee or institutional review board at each site and was conducted following Good Clinical Practice principles derived from the Declaration of Helsinki, and in accordance with local regulations and International Council for Harmonisation guidelines. All enrolled participants provided written informed consent.

### Evaluation of safety

The primary objective was to assess the long-term safety and tolerability of BUP/SAM. Study visits during the treatment phase occurred at weeks 1, 2, 4, 6, 8, 14, 20, 26, 32, 38, 44, and 52 as well as follow-up visits at weeks 53, 54, and 56.

Safety and tolerability assessments included treatment-emergent adverse events (AEs; screening and continuously at and between every study visit); vital signs (oral body temperature, respiratory rate, blood pressure, and pulse; screening and every study visit); weight (screening and every study visit); 12-lead electrocardiogram (ECG; screening and weeks 1, 26, 52, and 56); clinical laboratory parameters (chemistry, hematology, and urinalysis, including evaluation of hepatic effect in alanine aminotransferase [ALT] and aspartate aminotransferase [AST]; screening and weeks 1, 14, 26, 38, 52, and 56); Columbia-Suicide Severity Rating Scale (C-SSRS; screening and every study visit); and Clinical Opiate Withdrawal Scale (COWS; weeks 52 [baseline], 53, 54, and 56). Seven separate safety assessments were used to collect data by phone during the follow-up period, and if any AEs relating to opioid withdrawal were reported, the patient was asked to come into the clinic to receive a COWS assessment.

The C-SSRS is an evidence-supported suicidal ideation and behavior questionnaire with binary (yes/no) response categories. The percent of patients with “yes” answers to C-SSRS items and item shift from first the C-SSRS assessment were evaluated. The COWS is a 11-item questionnaire designed to measure the level of opiate withdrawal; a higher score indicates more severe signs and symptoms of withdrawal (0–4 = no withdrawal; 5–12 = mild; 13–24 = moderate; 25–36 = moderately severe; >36 = severe). COWS scores by mean days post last dose and a summary of COWS category shift from the first COWS assessment to the highest value were evaluated.

Serious AEs (SAEs) were defined as those resulting in death or immediate risk of death, inpatient hospitalization/prolonging of existing hospitalization, or disability/incapability. SAEs also included any congenital anomaly or important medical events that may not result in death, be immediately life threatening, or require hospitalization but may jeopardize the patient and/or require intervention to prevent one of the other outcomes listed above.

AEs of special interest were selected based on class effects that have been reported with BUP or SAM alone or with approved drugs for depression, including abuse potential, dependence, and opioid withdrawal, during the post-discontinuation period, hypomania/mania, sexual dysfunction, and suicidal ideation and behavior. To evaluate for abuse potential, preferred terms were selected according to FDA regulatory guidance and classified as related to abuse behavior, euphoria-related, or nonspecific in nature. The full list of preferred terms for each category of special interest is provided in the Supplementary Materials.

### Evaluation of efficacy

Exploratory outcomes included efficacy of up to 52 weeks of adjunctive BUP/SAM 2 mg/2 mg treatment. MADRS and the Clinical Global Impression of Severity (CGI-S) scores were measured at screening and every study visit through week 52. The 10-item MADRS questionnaire (with each item yielding a score between 0 and 6) and CGI-S scale (scored between 1 and 7) are each clinician administered, and higher scores indicate a more severe condition. The mean change from efficacy baseline (defined as initiation of BUP/SAM treatment) in MADRS and CGI-S scores at each study visit was reported. The proportion of patients who achieved remission (defined as MADRS ≤ 10) at each study visit and the time to remission were determined.

### Statistical methodology

Demographic and safety data were summarized using descriptive statistics. Demographics and baseline characteristics at study entry were collected at screening for de novo patients. Data from patients enrolled in one of the four prior studies were carried over from baseline of the prior study. To assess potential differences between patients with and without prior exposure to BUP/SAM, two subgroups were created: no prior exposure to BUP/SAM and prior exposure to BUP/SAM. Patients in the no prior exposure to BUP/SAM group included de novo patients and those who received placebo or no study drug in a prior BUP/SAM study. Patients in the prior exposure to the BUP/SAM group were those who completed one of the prior studies in one of the BUP/SAM treatment groups (BUP/SAM 0.5/0.5 mg, 1/1 mg, or 2/2 mg).

Safety was evaluated in all patients who received at least one dose of BUP/SAM in the long-term study (safety population). AEs, vital signs, laboratory analytes, and ECGs were tabulated and recorded throughout the long-term study. Patients meeting Temple’s corollary criteria (those with ALT or AST levels ≥3 times upper limit of normal range compared with placebo or nonhepatotoxic control drug) were tabulated [29]. Median duration was assessed for common AEs (those occurring in ≥5% of patients with or without prior BUP/SAM exposure). Safety baseline was defined as before or on the day of BUP/SAM initiation in the FORWARD-2 study. For C-SSRS shift analysis, patients were required to have a baseline and at least one post-baseline assessment. For analyses of dependence and withdrawal, patients were required to have ≥4 weeks’ exposure, a COWS baseline (the first COWS assessment after discontinuation of study drug) within 2 days of the last dose of the study drug, and at least one post-baseline COWS assessment.

Efficacy was explored in all patients who received at least one dose of BUP/SAM and had at least one post-baseline complete MADRS assessment in FORWARD-2; baseline was defined as the time of BUP/SAM initiation (in FORWARD-2 or in one of the prior short-term studies). Descriptive statistics with observed cases were used to assess mean MADRS, MADRS remission, and CGI-S scores by study visit.

The time to study discontinuation was estimated using Kaplan–Meier methods.

## Results

### Patients

Of the 1486 patients enrolled, 1485 entered the treatment phase of the study: 929 with no prior exposure to BUP/SAM, and 556 with prior exposure to BUP/SAM (Fig. S1). Patients were predominantly women (64.9%) and white (72.7%), with a mean age of 46.5 years; the majority were concurrently prescribed either SSRIs (62.3%) or SNRIs (26.6%) as their primary drug for depression (Table 1). Approximately 50% of enrolled patients completed the study (Fig. S2); withdrawal by patient (16.0%), AE (10.6%), and loss to follow-up (10.2%) were the most common reasons for discontinuation. Patients without prior BUP/SAM exposure discontinued due to AEs (13.1%) at twice the rate as those with prior BUP/SAM exposure (6.5%). The incidence of discontinuation for lack of efficacy was 4.2%. Of patients who entered the treatment phase, 978 (65.9%) had ≥6 months of BUP/SAM exposure and 769 (51.8%) had ≥12 months of exposure (including exposure during the prior studies) by the end of the long-term treatment period.

**Table 1:** Baseline^a^ demographic and clinical characteristics

Characteristic	No prior exposure to BUP/SAM (*n* = 929)	Prior exposure to BUP/SAM (*n* = 556)	All patients (*N* = 1485)
Age, years, mean (SD)	46.1 (12.4)	47.3 (12.1)	46.5 (12.3)
Sex, female, *n* (%)	616 (66.3)	348 (62.6)	964 (64.9)
Primary race, *n* (%)			
White	681 (73.3)	399 (71.8)	1080 (72.7)
Black or African American	224 (24.1)	138 (24.8)	362 (24.4)
American Indian or Alaska Native	3 (0.3)	4 (0.7)	7 (0.5)
Asian	15 (1.6)	13 (2.3)	28 (1.9)
Native Hawaiian or other Pacific Islander	6 (0.6)	2 (0.4)	8 (0.5)
Region, *n* (%)			
United States	735 (79.1)	469 (84.4)	1204 (81.1)
Non-United States	194 (20.9)	87 (15.6)	281 (18.9)
BMI (kg/m^2^), mean (SD)	29.6 (5.6)	29.4 (5.7)	29.5 (5.6)
MADRS total score, mean (SD)^b^	19.6 (9.8)	28.5 (6.4)	22.9 (9.7)
Duration of current MDE (months), mean (SD)	11.6 (8.6)	13.2 (5.5)	12.2 (7.6)
Class of drug for depression for current MDE, *n* (%)			
SSRI	566 (60.9)	359 (64.6)	925 (62.3)
SNRI	256 (27.6)	139 (25.0)	395 (26.6)
Bupropion	107 (11.5)	58 (10.4)	165 (11.1)
No. of inadequate responses for current MDE^c^, mean (SD)			
0	334 (36.0)	1 (0.2)^d^	335 (22.6)^d^
1	522 (56.2)	450 (80.9)^d^	972 (65.5)^d^
2+	73 (7.9)	67 (12.1)^d^	140 (9.4)^d^

*BMI* body mass index, *BUP* buprenorphine, *MADRS* Montgomery–Åsberg Depression Rating Scale, *MDE* major depressive episode, *SAM* samidorphan, *SD* standard deviation, *SNRI* serotonin–norepinephrine reuptake inhibitor, *SSRI* selective serotonin reuptake inhibitor

^a^Baseline was defined as time of BUP/SAM initiation (in FOWARD-2 or lead-in acute study); data shown are for patients who enrolled and had at least one dose of BUP/SAM in the FORWARD-2 study

^b^Baseline MADRS displayed for patients who also had at least one post-baseline assessment (*N* = 1453) and were included in efficacy analysis

^c^For de novo, this value is the number at visit 2 of FORWARD-2; for patients participating in the prospective lead-in period in the antecedent study (PLI failures), this value represents the number at the end of antecedent study; for patients who roll over from FORWARD-1, this value was not collected at randomization of antecedent study; for others, this value is the number at randomization of antecedent study

^d^The 38 patients in the prior exposure group who participated in FORWARD-1 have missing values for this variable, since it was not collected in the prior study

### Safety

AEs were reported by 1124 (75.7%) of the 1485 study participants (78.6% of no prior exposure to BUP/SAM group and 70.9% of prior exposure to the BUP/SAM group; Table 2). The majority (91.7%) of AEs reported were mild or moderate in severity. Severe events were reported by 123 patients (8.3%), and no specific severe event, as defined by a preferred term, was reported in ≥2% of patients. The most common AEs (occurring in ≥10% of patients) were nausea, headache, constipation, and dizziness (Table 2). Most common AEs were transient (median duration 1–2 weeks), except for constipation and dry mouth (median duration >5 weeks each) (Table S1). A total of 154 patients (10.4%) discontinued due to an AE (Table 2). AEs that led to discontinuation in ≥1% of patients were nausea, vomiting, and dizziness; although no tardive dyskinesia was reported, four (0.3%) patients discontinued due to tremor (Table S2).

**Table 2:** Summary of adverse events

Patients with event, *n* (%)	No prior exposure to BUP/SAM (*n* = 929)	Prior exposure to BUP/SAM (*n* = 556)	All patients (*N* = 1485)
Any AE	730 (78.6)	394 (70.9)	1124 (75.7)
Any SAE	33 (3.6)	14 (2.5)	47 (3.2)
Common SAEs (≥2 patients in any treatment group)			
Depression	2 (0.2)	1 (0.2)	3 (0.2)
Suicidal ideation	3 (0.3)	0	3 (0.2)
Colitis	2 (0.2)	0	2 (0.1)
Sepsis	2 (0.2)	0	2 (0.1)
Pneumonia	2 (0.2)	0	2 (0.1)
Uterine leiomyoma	2 (0.2)	0	2 (0.1)
Myocardial infarction	1 (0.1)	1 (0.2)	2 (0.1)
AE leading to study discontinuation	122 (13.1)	32 (5.8)	154 (10.4)
Common AEs (≥5% in any treatment group)			
Nausea	234 (25.2)	88 (15.8)	322 (21.7)
Headache	103 (11.1)	53 (9.5)	156 (10.5)
Constipation	112 (12.1)	39 (7.0)	151 (10.2)
Dizziness	116 (12.5)	34 (6.1)	150 (10.1)
Somnolence	100 (10.8)	24 (4.3)	124 (8.4)
Vomiting	83 (8.9)	33 (5.9)	116 (7.8)
Dry mouth	66 (7.1)	21 (3.8)	87 (5.9)
Fatigue	62 (6.7)	23 (4.1)	85 (5.7)
Upper respiratory tract infection	50 (5.4)	33 (5.9)	83 (5.6)
Insomnia	55 (5.9)	26 (4.7)	81 (5.5)
Nasopharyngitis	46 (5.0)	34 (6.1)	80 (5.4)
Sedation	51 (5.5)	15 (2.7)	66 (4.4)
Hyperhidrosis	48 (5.2)	9 (1.6)	57 (3.8)

AEs were coded by preferred terms and system organ class using the *Medical Dictionary for Regulatory Activities*, version 19.0

*AE* adverse event, *BUP* buprenorphine, *SAE* serious adverse event, *SAM* samidorphan

SAEs were reported in 47 patients (3.2%) (Table 2 [common SAEs]; Table S3 [complete listing]). There was no identifiable pattern of events, and no particular SAE by preferred term occurred in >3 (0.2%) patients. Two deaths were reported during the study, both in the no prior exposure to the BUP/SAM group—respiratory arrest in a reportedly nonadherent patient with undisclosed chronic obstructive pulmonary disease and cerebral hemorrhage in a patient with medical and familial risk factors. Both deaths were deemed unrelated to study drug by the investigators and were reported to the relevant IRB. Full details of the patient deaths can be found in the Supplementary Results.

There were no completed suicides during the study. Seven patients (0.5%) reported suicidal ideation events, and no other AE potentially associated with suicidal ideation or behavior was reported. At baseline, C-SSRS suicidal ideation was reported for 4.8% of patients, and no C-SSRS suicidal behavior was reported in any patient. At any post-baseline visit, C-SSRS suicidal ideation and behavior, respectively, were reported in 153 (10.3%) and one (0.1%) patient; at the last visit, 53 (3.6%) and one (0.1%). One hundred patients (6.8%) reported an increase in C-SSRS suicidal ideation from baseline; only one patient without suicidal ideation at baseline reported C-SSRS serious suicidal ideation during treatment (Table S4). Of the 71 patients who reported any C-SSRS suicidal ideation at baseline, 58 (81.7%) improved during treatment. Of those with post-baseline C-SSRS suicidal ideation, only four patients (0.2%) reported active ideation (two with some intent to act without a plan and two with a specific plan and intent).

The incidence of any AE potentially associated with sexual dysfunction was low (1.8%). Three patients (0.2%) discontinued due to sexual dysfunction-related AEs (decreased libido: two [concomitant SSRI and concomitant SNRI, respectively]; ejaculation failure: one [concomitant SNRI]). There was no evidence of increased risk of hypomania/mania based on evaluation of clustering of AEs potentially associated with hypomania/mania (i.e., ≥3 AEs of this class in a single patient).

AEs potentially associated with abuse potential were experienced by 321 patients (21.6%) (Table 3). The majority of these events (98.1%; 315/321) were nonspecific in nature (e.g., dizziness, somnolence, and other AEs potentially associated with abuse potential, but which also occur in drugs with no abuse potential). Euphoria-related events were infrequent and were reported in 18 patients (1.2%). All were single events, and none reoccurred. There were no AEs potentially associated with abuse behavior (e.g., intentional overdose, product tampering) or dependence (e.g., drug dependence antepartum/postpartum).

**Table 3:** Adverse events to evaluate for abuse potential

Patients with event, *n* (%)	No prior exposure to BUP/SAM (*n* = 929)	Prior exposure to BUP/SAM (*n* = 556)	All patients (*N* = 1485)
Any AESI to evaluate abuse potential	248 (26.7)	73 (13.1)	321 (21.6)
Euphoria related	16 (1.7)	2 (0.4)	18 (1.2)
Feeling abnormal	7 (0.8)	2 (0.4)	9 (0.6)
Euphoric mood	6 (0.6)	0	6 (0.4)
Feeling drunk	2 (0.2)	0	2 (0.1)
Hallucination, auditory	1 (0.1)	0	1 (0.1)
Abuse behavior	0	0	0
Abuse potential nonspecific	242 (26.0)	73 (13.1)	315 (21.2)
Dizziness	116 (12.5)	34 (6.1)	150 (10.1)
Somnolence	100 (10.8)	24 (4.3)	124 (8.4)
Sedation	51 (5.5)	15 (2.7)	66 (4.4)
Disturbance in attention	9 (1.0)	4 (0.7)	13 (0.9)
Confusional state	2 (0.2)	0	2 (0.1)
Cognitive disorder	0	1 (0.2)	1 (0.1)
Disorientation	1 (0.1)	0	1 (0.1)
Dissociation	1 (0.1)	0	1 (0.1)
Mood swings	1 (0.1)	0	1 (0.1)
Paranoia	1 (0.1)	0	1 (0.1)

Adverse events were coded by preferred terms and system organ class using the *Medical Dictionary for Regulatory Activities*, version 19.0. A full list of preferred terms evaluated are in the Supplementary Materials

*AESI* adverse event of special interest, *BUP* buprenorphine, *SAM* samidorphan

Following BUP/SAM discontinuation, fewer than 10% of patients reported AEs potentially associated with opioid withdrawal (Table 4). The most common of these AEs (≥1% of patients) were insomnia (2.0%), headache (1.6%), diarrhea (1.2%), and anxiety (1.1%). Of the nine patients who had ≥3 events possibly related to withdrawal, only one received prescription treatment for symptoms. There were four (0.4%) reported cases of drug-withdrawal syndrome. The highest post-discontinuation COWS scores among these four patients were categorized as no withdrawal (*n* = 2) and mild withdrawal (*n* = 1); one patient had no COWS score recorded. No opioids were reported as prior or concomitant medication for any of these four patients. The drug-withdrawal syndrome event resolved without treatment in all but one of these four patients (who was treated with clonidine 0.1 mg twice daily, until the event resolved in 11 days). Full details for these four patients can be found in the Supplementary Results.

**Table 4:** Post-discontinuation adverse events to evaluate opioid withdrawal potential

Patients with event, *n* (%)	All patients (*N* = 1109)
Any PDAE associated with opioid withdrawal	109 (9.8)
PDAEs associated with opioid withdrawal occurring in ≥3 patients	
Insomnia	22 (2.0)
Headache	18 (1.6)
Diarrhea	13 (1.2)
Anxiety	12 (1.1)
Irritability	10 (0.9)
Nausea	9 (0.8)
Rhinorrhea	9 (0.8)
Hyperhidrosis	8 (0.7)
Pain	8 (0.7)
Restlessness	8 (0.7)
Arthralgia	6 (0.5)
Drug withdrawal syndrome	4 (0.4)
Yawning	4 (0.4)
Chills	3 (0.3)

Adverse events were coded by preferred terms and system organ class using the *Medical Dictionary for Regulatory Activities*, version 19.0. A full list of preferred terms evaluated are in the Supplementary Materials

*PDAE* post-discontinuation adverse event

In the overall population, mean COWS scores were <1.0 at every assessment throughout the 4-week follow-up period, and the mean change from the post-discontinuation baseline at each follow-up visit was <1.0 (Table S5). COWS scores ≤ 4 are categorized as no withdrawal. The incidence of COWS score categorical worsening from no withdrawal at baseline was low (*n* = 58; 6.5%). Of these 58 cases, 49 were categorized as mild withdrawal (5.5%) and the remaining nine were moderate (1.0%). None of the patients who worsened from no withdrawal to moderate withdrawal required prescription treatment, and of those who worsened to mild withdrawal, only three required treatment (benzodiazepine for sleep and/or anxiety). No patient experienced a worsening from no withdrawal at baseline to moderately severe or severe withdrawal.

BUP/SAM was not associated with meaningful changes in weight during the study (Table S6). A total of 23 unique patients (1.6%) met Temple’s corollary criteria. There were no other clinically meaningful mean changes from baseline in lipids or other chemistry analytes, hematology laboratory analyses, or urinalyses (Table S7). Mean changes from baseline in heart rate, blood pressure, respiratory rate, and temperature were within the normal range. There were no potentially clinically significant changes in vital signs or body weight at the last visit (Table S8). No changes in ECG records were deemed clinically significant.

### Exploratory evaluation of efficacy

**Fig. 1:**
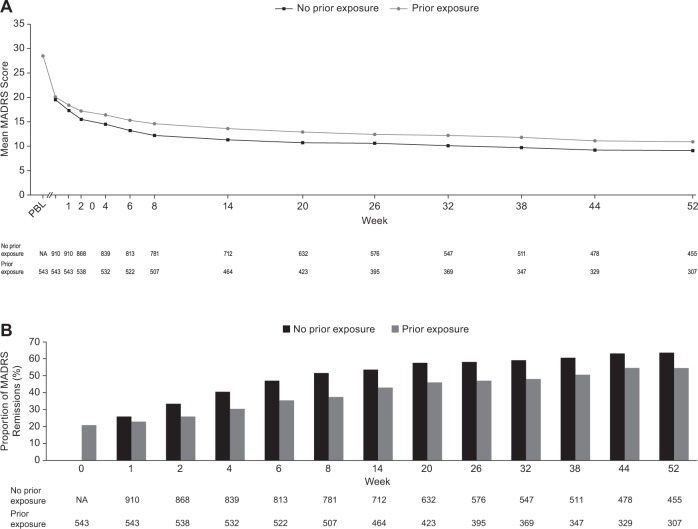
**a** Mean MADRS scores over time and **b** proportion of patients achieving remission during BUP/SAM treatment. Data were based on observed cases. Week 0 refers to baseline of this study and was the baseline for patients without prior BUP/SAM exposure. PBL refers to the prior baseline for those patients with prior BUP/SAM exposure, i.e., their baseline from study FORWARD-1, FORWARD-3, FORWARD-4, or FORWARD-5. Each subsequent week refers to week of study visit during FORWARD-2 and corresponds to week since BUP/SAM initiation for patients with no prior BUP/SAM exposure. Remission was defined as Montgomery–Åsberg Depression Rating Scale score ≤ 10. *BUP/SAM* buprenorphine/samidorphan, *MADRS* Montgomery–Åsberg Depression Rating Scale, *PBL* prior baseline

MADRS scores showed sustained improvement in those patients continuing BUP/SAM treatment, persisting until the end of the 52-week study (Fig. 1a). More than half of all patients (60.2%) were in remission at the last study visit (55.0 and 63.7% of those with and without prior exposure to BUP/SAM, respectively) (Fig. 1b). The median time to remission was 99.0 days from study drug initiation. CGI-S scores improved during long-term BUP/SAM treatment, with a mean change from baseline at the last study assessment of –1.0 (SD ± 1.3).

## Discussion

In the 52-week FORWARD-2 study, adjunctive treatment with BUP/SAM 2 mg/2 mg was generally well tolerated, with no new or unexpected safety signals arising from long-term treatment. BUP/SAM demonstrated low risk of abuse. There was little evidence of withdrawal upon abrupt discontinuation, as assessed by AEs and COWS, and there was low incidence of other AEs commonly associated with drugs for depression and adjunctive agents used in the treatment of MDD.

MDD is a chronic condition for which long-term treatment is recommended [30–32]. Currently approved drugs for psychosis used as adjunctive treatment of MDD belong to a single class. Given that BUP/SAM has a novel mechanism of action, its favorable tolerability and safety profile over 52 weeks is noteworthy. The AE profile in FORWARD-2 was consistent with that reported in prior short-term, placebo-controlled studies of BUP/SAM. There were no suicide attempts during the treatment period, nor was there any evidence of increased risk for suicidal ideation or behavior with BUP/SAM [24, 27, 28]. AEs were predominantly gastrointestinal and sedation-related events, occurring with frequency similar to those observed in long-term studies of approved monotherapies and adjunctive agents [33–36]. In addition, the AE rates were higher in patients starting treatment in this study than in those who participated in prior BUP/SAM studies, indicating that events are associated with initiation. The rate of discontinuation due to AEs was 10.6%, which is also consistent with rates reported with approved drugs for depression. In long-term MDD studies of adjunctive aripiprazole and brexpiprazole, the rates of discontinuation due to AEs were 23.0% and 14.1%, respectively [10, 37, 38]. The discontinuation rates reported with up to 14 weeks of treatment with adjunctive lithium and triiodothyronine (T_3_) were 23.0 and 9.6%, respectively [39]. The discontinuation rates observed for open-label extension studies with monotherapies, such as duloxetine, escitalopram, and vortioxetine range from 6 to 12% [33, 35, 36, 40].

Adjunctive atypical drugs for psychosis are commonly associated with weight gain and adverse metabolic changes [10, 11]. Adjunctive aripiprazole has been associated with akathisia (number needed to harm [NNH]: 4) and significant weight gain (≥7% from baseline; NNH: 29). Weight gain has also been associated with adjunctive olanzapine/fluoxetine (≥10% from baseline; NNH: 9) and adjunctive quetiapine (≥7% from baseline; NNH: 37). Weight remained stable with long-term adjunctive BUP/SAM treatment and BUP/SAM was not associated with clinically meaningful changes in metabolic parameters, vital signs, laboratory tests, or ECG parameters. No akathisia was reported, and although there was low incidence (*n* = 4) of discontinuation due to tremor, no tardive dyskinesia was reported.

BUP and other μ-opioid receptor agonists have shown antidepressant potential in the treatment of MDD [19, 20, 25, 41], but the established abuse liability of μ-opioid receptor agonists limits their utility [19, 41, 42]. SAM was included in the BUP/SAM combination to mitigate the risk of abuse and dependence associated with BUP [22]. The results of FORWARD-2 are consistent with those of the short-term studies of BUP/SAM treatment of patients with MDD, providing empirical evidence of the safety and tolerability for longer-term BUP/SAM therapy. The incidence of euphoria-related events was low and there was no indication of abuse behavior or dependence as assessed by AEs and investigator report; most of the AEs reported to evaluate for abuse potential were nonspecific (i.e., dizziness and somnolence). These results support and extend the finding of reduced abuse potential of BUP/SAM compared with BUP demonstrated in a human abuse potential study [26] by providing evidence that SAM mitigated the abuse potential associated with BUP over a 1-year period in patients with MDD.

There was little evidence of withdrawal after abrupt discontinuation of BUP/SAM, as evidenced by AE and COWS assessments. Withdrawal is associated with abrupt discontinuation of many commonly used antidepressants [43–45]. Abrupt discontinuation of BUP alone is associated with mild-to-moderate opioid withdrawal in almost all patients and usually requires symptomatic management of opioid withdrawal [46–49]. As abrupt discontinuation of BUP/SAM after up to 52 weeks of treatment was well tolerated, this differentiates BUP/SAM from unmitigated µ-opioid receptor agonists and further indicates that SAM attenuated the μ-agonist activity of BUP, precluding the development of physical dependence associated with BUP alone.

An exploratory assessment of efficacy found an antidepressant effect with demonstrated durability for up to 52 weeks. These data support the efficacy findings from short-term, placebo-controlled studies [24, 27, 28] covering treatment in more than 700 patients over a full year. The improvement in mean MADRS scores was sustained among patients who continued BUP/SAM treatment for up to 52 weeks. In addition, ∼60% of patients continuing treatment achieved remission by study end. The rate of remission is comparable to that seen with other adjunctive treatments for patients with MDD who are inadequate responders to first-line antidepressant therapy [50, 51]. This  is promising, given the favorable long-term safety and tolerability results. The baseline MADRS scores for patients with prior BUP/SAM exposure, which represent the baseline scores from the prior study, were higher than baseline MADRS scores of patients in this study without prior BUP/SAM exposure, because the inclusion criteria for FORWARD-1, FORWARD-3, FORWARD-4, and FORWARD-5 required a more severe depression rating at enrollment than FORWARD-2. This also may have contributed to the slight difference in remission rate between the two groups, because patients without prior BUP/SAM exposure who entered the study with lower MADRS scores could more easily attain a MADRS score ≤ 10.

Findings should be interpreted with consideration of the study’s limitations. The open-label design and lack of control group limit the ability to attribute AEs solely to adjunctive BUP/SAM treatment, because some AEs may have resulted from the long-term use of background drugs for depression and/or the natural course of the illness. In addition, the clinically meaningful reduction in MADRS scores and achievement of remission by ~60% of study completers must be seen in the context of both a lack of a control arm and the dropout rate. With nearly half of study participants dropping out, it is possible that nonremitters dropped out at a greater rate than remitters. However, the overall dropout rate due to lack of efficacy was low (4.2%), and the study findings are strengthened by the large number and diverse background of patients with MDD who received BUP/SAM over 1 year.

The opioid system modulating BUP/SAM combination represents a promising potential adjunctive treatment that works primarily via a nonaminergic mechanism for patients with MDD who do not respond adequately to drugs for depression. Consistent with its intended effect, SAM mitigated the abuse potential of BUP, with the combination exhibiting little evidence of abuse potential or withdrawal symptoms after a year of treatment. Moreover, treatment with BUP/SAM was generally well tolerated; the safety profile was consistent with that observed in placebo-controlled studies, including a favorable profile for suicidal ideation and behavior. Further, durability of therapeutic effect was observed with long-term treatment. These data suggest that BUP/SAM may help to address the critical need for new treatments capable of providing long-term benefit for patients with MDD.

## Funding and disclosure

The study, including study design, collection, analysis, and interpretation of data, was sponsored by Alkermes, Inc. The Perelman School of Medicine of the University of Pennsylvania received a grant from Assurex so that Dr. Thase could conduct the research protocol described in this report at his site. Dr. Thase has served as an advisor or consultant to Acadia, Akilii, Alkermes, Inc, Allergan (includes Forest Laboratories and Naurex), AstraZeneca, Cerecor, Eli Lilly, Fabre-Kramer, Gerson Lehrman Group, Guidepoint Global, Johnson & Johnson Pharmaceutical Research & Development LLC (Janssen, Ortho-McNeil), Lundbeck, MedAvante, Merck, Moksha8, Nestlé (PamLab), Novartis, Otsuka, Pfizer, Shire, Sunovion, and Takeda; he has received grant support from Acadia, Agency for Healthcare Research and Quality, Alkermes, Inc, Avanir, Forest, Intracellular, Janssen, National Institute of Mental Health, Otsuka, Patient Centered Outcomes Research Institute, and Takeda; he has received royalties from American Psychiatric Press, Guilford Publications, Herald House, and WW Norton & Company Inc; and Dr Thase’s spouse is employed by Peloton Advantage, which does business with a number of pharmaceutical companies. Dr. Bodkin has served as an advisor to Alkermes, Inc; he has received research support from McLean Hospital via Alkermes, Inc. Dr. Trivedi has served as an advisor or consultant to AcademyHealth, Alkermes, Inc, Akili Interactive, Allergan Pharmaceuticals, ACADIA Pharmaceuticals Inc, American Society of Clinical Psychopharmacology, Brain Institute Canada (CAN-BIND), Brintellix Global, Global Medical Education, Healthcare Global Village, Health Research Associates, Jazz Pharmaceuticals, Lundbeck Research USA, Medscape LLC, MSI Methylation Sciences Inc, Nestlé Health Science—Pamlab Inc, Naurex Inc, Navitor, One Carbon Therapeutics, Otsuka America Pharmaceutical Inc, Saatchi, and Takeda Global Research; he has received grant support from National Institute of Mental Health, National Institute on Drug Abuse, Johnson & Johnson Pharmaceutical Research & Development LLC, and Janssen Research & Development LLC; he has received royalties from Janssen Research & Development LLC; he has publications for Janssen Asia Pacific and Oxford University Press. Dr. Fava has received research support from Abbott Laboratories, Acadia Pharmaceuticals, Alkermes, Inc, American Cyanamid, Aspect Medical Systems, AstraZeneca, Avanir Pharmaceuticals, AXSOME Therapeutics, BioResearch, BrainCells Inc; Bristol-Myers Squibb, CeNeRx BioPharma, Cephalon, Cerecor, Clintara, LLC, Covance, Covidien, Eli Lilly & Co, EnVivo Pharmaceuticals Inc, Euthymics Bioscience Inc, Forest Pharmaceuticals Inc, FORUM Pharmaceuticals, Ganeden Biotech Inc, GlaxoSmithKline, Harvard Clinical Research Institute, Hoffman-LaRoche, i3 Innovus/Ingenix, Icon Clinical Research, Janssen R&D LLC, Jed Foundation, Johnson & Johnson Pharmaceutical Research & Development LLC, Lichtwer Pharma GmbH, Lorex Pharmaceuticals, Lundbeck Inc, Marinus Pharmaceuticals, MedAvante, Methylation Sciences Inc, National Alliance for Research on Schizophrenia & Depression, National Center for Complementary and Alternative Medicine, National Coordinating Center for Integrated Medicine, National Institute of Drug Abuse, National Institute of Mental Health, Neuralstem Inc, NeuroRx, Novartis AG, Organon Pharmaceuticals, Otsuka Pharmaceutical Development Inc, PamLab LLC, Pfizer Inc, Pharmacia-Upjohn, Pharmaceutical Research Associates Inc, Pharmavite LLC, PharmoRx Therapeutics, Photothera, RCT Logic LLC (formerly Clinical Trials Solutions LLC), Reckitt Benckiser, Roche Pharmaceuticals, Sanofi-Aventis US LLC, Shire, Solvay Pharmaceuticals Inc, Stanley Medical Research Institute, Synthelabo, Taisho Pharmaceuticals, Takeda Pharmaceuticals, Tal Medical, VistaGen, and Wyeth-Ayerst Laboratories; he has served as an advisor or consultant to Abbott Laboratories, Acadia, Affectis Pharmaceuticals AG, Alkermes, Inc, Amarin Pharma Inc, Aspect Medical Systems, AstraZeneca, Auspex Pharmaceuticals, Avanir Pharmaceuticals, AXSOME Therapeutics, Bayer AG, Best Practice Project Management Inc, Biogen, BioMarin Pharmaceuticals Inc, Biovail Corporation, BrainCells Inc, Bristol-Myers Squibb, CeNeRx BioPharma, Cephalon Inc, Cerecor, CNS Response Inc, Compellis Pharmaceuticals, Cypress Pharmaceutical Inc, DiagnoSearch Life Sciences (P) Ltd, Dinippon Sumitomo Pharma Co Inc, Dov Pharmaceuticals Inc, Edgemont Pharmaceuticals Inc, Eisai Inc, Eli Lilly & Co, EnVivo Pharmaceuticals Inc, ePharmaSolutions, EPIX Pharmaceuticals Inc, Euthymics Bioscience Inc, Fabre-Kramer Pharmaceuticals Inc, Forest Pharmaceuticals Inc, Forum Pharmaceuticals, GenOmind LLC, GlaxoSmithKline, Grunenthal GmbH, i3 Innovus/Ingenis, Indivior, Intracellular, Janssen Pharmaceuticals, Jazz Pharmaceuticals Inc, Johnson & Johnson Pharmaceutical Research & Development LLC, Knoll Pharmaceuticals Corp, Labopharm Inc, Lorex Pharmaceuticals, Lundbeck Inc, Marinus Pharmaceuticals, MedAvante Inc, Merck & Co Inc, MSI Methylation Sciences Inc, Naurex Inc, Navitor Pharmaceuticals Inc, Nestlé Health Sciences, Neuralstem Inc, Neuronetics Inc, NextWave Pharmaceuticals, Novartis AG, Nutrition 21, Orexigen Therapeutics Inc, Organon Pharmaceuticals, Osmotica, Otsuka Pharmaceuticals, Pamlab LLC, Pfizer Inc, PharmaStar, Pharmavite LLC, PharmoRx Therapeutics, Precision Human Biolaboratory, Prexa Pharmaceuticals Inc PPD, PsychoGenics, Psylin Neurosciences Inc, Purdue Pharma, Puretech Ventures, RCT Logic LLC (formerly Clinical Trials Solutions LLC), Relmada Therapeutics Inc, Rexahn Pharmaceuticals Inc, Ridge Diagnostics Inc, Roche Pharmaceuticals, Sanofi-Aventis US LLC, Sepracor Inc, Servier Laboratories, Schering-Plough Corporation, Shenox Pharmaceuticals, Solvay Pharmaceuticals Inc, Somaxon Pharmaceuticals Inc, Somerset Pharmaceuticals Inc, Sunovion Pharmaceuticals, Supernus Pharmaceuticals Inc, Synthelabo, Taisho Pharmaceuticals, Takeda Pharmaceutical Co Ltd, Tal Medical Inc, Tetragenex, Teva Pharmaceuticals, TransForm Pharmaceuticals Inc, Transcept Pharmaceuticals Inc, Usona Institute Inc, Vanda Pharmaceuticals Inc, Versant Venture Management LLC, and VistaGen; he has had speaking or publishing roles for Adamed Co, Advanced Meeting Partners, American Psychiatric Association, American Society of Clinical Psychopharmacology, AstraZeneca, Belvoir Media Group, Boehringer Ingelheim GmbH, Bristol-Myers Squibb, Cephalon Inc, CME Institute/Physicians Postgraduate Press, Inc, Eli Lilly & Co, Forest Pharmaceuticals Inc, GlaxoSmithKline, Imedex LLC, MGH Psychiatry Academy/Primedia, Massachusetts General Hospital (MGH) Psychiatry Academy/Reed Elsevier, Novartis AG, Organon Pharmaceuticals, Pfizer Inc, PharmaStar, United BioSource Corp, and Wyeth-Ayerst Laboratories; he is named on patents for sequential parallel comparison design, licensed by MGH to Pharmaceutical Product Development, and pharmacogenomics of depression treatment with folate; he has a patent application for a combination of ketamine plus scopolamine in major depressive disorder, licensed by MGH to Biohaven; he is a copyright holder for the MGH Cognitive & Physical Functioning Questionnaire, Sexual Functioning Inventory, Antidepressant Treatment Response Questionnaire, Discontinuation-Emergent Signs & Symptoms, Symptoms of Depression Questionnaire, and SAFER; he has publications for Lippincott, Williams & Wilkins, Wolkers Kluwer, and World Scientific Publishing. Drs. Stanford, Memisoglu, Martin, Claxton, Yu, and Pathak are employees and stockholders of Alkermes, Inc.

## Supplementary information

Supplemental Material

Supplemental Figure 1

Supplemental Figure 2

## Data Availability

All data on which conclusions of the paper rely are available within the main text and/or Supplementary Materials.
