# Supplementary material for: Results from a long-term open-label extension study of adjunctive buprenorphine/samidorphan combination in patients with major depressive disorder
Source: Neuropsychopharmacology. 2019 Jun 29;44(13):2268–76. doi: 10.1038/s41386-019-0451-3 (PMC6897901; doi:10.1038/s41386-019-0451-3)
Supplement: Supplementary file 1 — Supplemental Material [file 41386_2019_451_MOESM1_ESM.docx]

Results from a long-term open-label extension study of adjunctive buprenorphine/samidorphan combination in patients with major depressive disorder

Michael E. Thase, MD^1^^[[1]](#footnote-1)^*, Arielle D. Stanford, MD^2^, Asli Memisoglu, ScD^2^, William Martin, PhD^2^, Amy Claxton, PhD^2^, J. Alexander Bodkin, MD^3,4^, Madhukar H. Trivedi, MD^5^, Maurizio Fava, MD^4,6^, Miao Yu, PhD^2^ and Sanjeev Pathak, MD^2^

^1^University of Pennsylvania School of Medicine, Philadelphia, PA, USA; ^2^Alkermes, Inc., Waltham, MA, USA; ^3^McLean Hospital, Belmont, MA, USA; ^4^Harvard Medical School, Boston, MA, USA; ^5^University of Texas Southwestern Medical Center, Dallas, TX, USA and ^6^Massachusetts General Hospital, Boston, MA, USA

*Correspondence to: Perelman School of Medicine, Department of Psychiatry, 3535 Market Street, Suite 670, Philadelphia, PA 19104-3309, USA.
Tel: 215-746-6680 / Fax: 215-573-0759
*E-mail address*: thase@mail.med.upenn.edu (M.E. Thase)

Supplementary Materials^[[2]](#footnote-2)^

**Supplementary Methods:** The supplementary Methods section contains the full inclusion/exclusion criteria, additional eligibility and screening details, and the complete list of coded/preferred terms for adverse events of special interest that were used to evaluate the potential for abuse, suicidal ideation, sexual dysfunction, the potential for opioid withdrawal, and the potential for dependence.

**Supplementary Results:** The supplementary textual Results section includes full patient details for the two patients who died during the study as well as full patient details for the four patients who experienced drug withdrawal syndrome.

Content overview

[**SUPPLEMENTARY METHODS**](#_Toc533767527) 3

[**SUPPLEMENTARY RESULTS**](#_Toc533767528) 4

**Table S1.** Duration of common adverse events……………………………………………………………………………………….6

[**Table S2.** Adverse events leading to discontinuation 7](#_Toc533767529)

[**Table S3.** Serious adverse events reported during the treatment period in ≥1 patient in any treatment group 8](#_Toc533767530)

[**Table S4.** Suicide-related events based on Columbia Suicide Severity Rating Scale (C-SSRS) during treatment period 10](#_Toc533767531)

[**Table S5.** Clinical Opiate Withdrawal Scale (COWS) score by time interval during postdiscontinuation safety period 11](#_Toc533767532)

[**Table S6.** Patients with potentially clinically significant values in weight at and after baseline assessment during treatment period 13](#_Toc533767533)

**Table S7.** Patients with potentially clinically significant values in cholesterol at last visit……………………….14

[**Table S8.** Patients with potentially clinically significant values in vital signs or body weight at last visit 15](#_Toc533767534)

[**Fig. S1.** Patient disposition. 16](#_Toc533767535)

**Fig. S2.** Patient retention over time……………………………………………………………………………………………………….17

**SUPPLEMENTARY METHODS**

Patient enrollment

Patients from FORWARD-3, FORWARD-4, and FORWARD-5 who demonstrated response (>50% reduction in depressive symptom severity to an adequate dose for ≥8 weeks) but not remission (MADRS <10) after completing the prospective lead-in periods (and thus not randomized) were only eligible to enroll in FORWARD-2 within 10 days of their final visit. If >10 days had elapsed since the final visit of the last study, these patients were screened for eligibility alongside *de novo* patients.

Adverse events (AEs) of special interest

AEs were coded by preferred terms and system organ class using the *Medical Dictionary for Regulatory Activities*, version 19.0. The full list of preferred terms for each category of special interest are as follows:

- Suicidal ideation and/or behavior: completed suicide, depression suicidal, intentional overdose, intentional self-injury, poisoning deliberate, self-injurious behavior, self-injurious ideation, suicidal behavior, suicidal ideation, suicide attempt.
- Sexual dysfunction: decreased libido, erectile dysfunction, ejaculation delayed, sexual dysfunction, loss of libido, abnormal orgasm, disturbance in sexual arousal, ejaculation failure.
- Abuse potential:
  - Abuse behavior related: accidental overdose, drug abuser, drug diversion, drug level above therapeutic, drug level increased, drug screen, drug screen positive, intentional overdose, intentional product misuse, intentional product use issue, maternal use of illicit drugs, needle track marks, neonatal complications of substance abuse, overdose, prescription drug used without a prescription, prescription form tampering, product tampering, substance abuse, substance abuser, substance use, substance-induced mood disorder, substance-induced psychotic disorder, toxicity to various agents.
  - Nonspecific: acute psychosis, aggression, cognitive disorder, confusional state, delirium, delusional disorder, unspecified type, depersonalization/derealization disorder, disorientation, dissociation, disturbance in attention, disturbance in social behavior, dizziness, dopamine dysregulation syndrome, emotional disorder, flight of ideas, medication overuse headache, mental impairment, mood altered, mood swings, narcotic bowel syndrome, paranoia, psychotic behavior, psychotic disorder, sedation, somnolence, stupor.
  - Euphoria-related: euphoric mood, feeling abnormal, feeling drunk, feeling of relaxation, hallucination (auditory, gustatory, mixed, olfactory, synesthetic, tactile, visual), inappropriate affect, thinking abnormal.
- Potential dependence: drug dependence, drug dependence antepartum/postpartum, drug tolerance, drug tolerance decreased/increased, substance dependence.
- Potential opioid withdrawal: drug detoxification, reversal of opiate activity, drug rehabilitation, drug withdrawal convulsions, drug withdrawal headache, drug withdrawal maintenance therapy, drug withdrawal syndrome, drug withdrawal syndrome neonatal, rebound effect, steroid withdrawal syndrome, withdrawal arrhythmia, withdrawal syndrome, anhedonia, depressed mood, depression, dysphoria, feeling of despair, morose, negative thoughts, persistent depressive disorder, dyssomnia, headache, insomnia, obsessive thoughts, poor quality sleep, syncope, terminal insomnia, agitation, irritability, anxiety, chills, hyperhidrosis, nausea, nervousness, pain, tremor, vomiting, abdominal pain, arthralgia, diarrhea, mydriasis, piloerection, restlessness, rhinorrhea, tachycardia, yawning.

**SUPPLEMENTARY RESULTS**

Patient deaths

Two patient deaths occurred during the study; both were deemed unrelated to study drug by the investigator.

- Patient 1 died from respiratory arrest 47 days after the last reported dose of BUP/SAM and had chronic obstructive pulmonary disease based on post-event medical history. He was taking a concomitant nonbenzodiazepine benzodiazepine-receptor agonist known to be associated with respiratory depression and was identified as being nonadherent with BUP/SAM.
- Patient 2 died from cerebral hemorrhage after 87 days of treatment during FORWARD-2 and had a parent who died from the same cause. She was an ex-smoker with a history of hypertension and, based on post-event medical history, pulmonary congestion, left ventricular hypertrophy, arterial hypertension, a “hypertensive heart,” and chronic heart failure functional class II.

Cases of drug withdrawal syndrome

- **Case 1** was in a 47-year-old female taking escitalopram 20 mg daily background drug for depression therapy with prior exposure to BUP/SAM; opioid withdrawal syndrome was reported 3 days post–last dose of BUP/SAM, and included reports of anxiety, hand tremors, headache, excessive sweating, restlessness, and diminished energy. The patient’s highest postdiscontinuation COWS score was 7 (mild withdrawal, reported during time of AE). No treatment was reported, and the event resolved in 41 days.
- **Case 2** was in a 59-year-old female taking citalopram 20 mg daily background drug for depression with no prior BUP/SAM who experienced opioid withdrawal symptoms 3 days following the last dose of BUP/SAM; the symptoms reported were restlessness, anxiety, and nausea. The patient’s highest postdiscontinuation COWS score was 2 (no withdrawal). The patient was treated with clonidine 0.1 mg twice daily, and the event resolved in 11 days.
- **Case 3** was in a 65-year-old male taking escitalopram 20 mg daily background drug for depression with prior exposure to BUP/SAM who experienced opioid withdrawal syndrome 8 days after the last dose of BUP/SAM; restlessness and feeling achy were the only symptoms reported. The patient’s highest postdiscontinuation COWS score was 0 (no withdrawal). No treatment was reported, and the event resolved the following day.
- **Case 4** was in a 60-year-old female taking escitalopram 10 mg daily background drug for depression who had no prior exposure to BUP/SAM and experienced general drug withdrawal symptoms 2 days following the last dose of BUP/SAM; symptoms reported were “electrical” and “sizzling” sounds in her head (a description more consistent with SSRI/SNRI withdrawal than opioid withdrawal). The patient had no postdiscontinuation COWS score recorded. No treatment was reported, and the event resolved in 11 days.

**Table S1.** Duration of common adverse events

| **Preferred term  statistics** | **No prior exposure  to BUP/SAM (*n* = 929)** | **Prior exposure  to BUP/SAM (*n* = 556)** |
| --- | --- | --- |
| Nausea, *n*^a^ | 222 | 83 |
| Median days (P10, P90) | 10.0 (2.0, 88.0) | 23.0 (2.0, 250.0) |
| Days (min, max) | (1, 396) | (1, 368) |
| Headache, *n*^a^ | 96 | 49 |
| Median days (P10, P90) | 8.5 (1.0, 65.0) | 8.0 (1.0, 163.0) |
| Days (min, max) | (1, 396) | (1, 359) |
| Dizziness, *n*^a^ | 110 | 30 |
| Median days (P10, P90) | 8.0 (1.0, 53.0) | 6.0 (1.0, 119.0) |
| Days (min, max) | (1, 357) | (1, 362) |
| Nasopharyngitis, *n*^a^ | 46 | 34 |
| Median days (P10, P90) | 9.0 (4.0, 29.0) | 7.0 (4.0, 17.0) |
| Days (min, max) | (2, 58) | (2, 47) |
| Constipation, *n*^a^ | 101 | 34 |
| Median days (P10, P90) | 35.0 (7.0, 340.0) | 44.5 (8.0, 316.0) |
| Days (min, max) | (1, 376) | (4, 365) |
| Vomiting, *n*^a^ | 83 | 32 |
| Median days (P10, P90) | 2.0 (1.0, 17.0) | 2.0 (1.0, 20.0) |
| Days (min, max) | (1, 348) | (1, 65) |
| Upper respiratory tract infection, *n*^a^ | 48 | 31 |
| Median days (P10, P90) | 10.5 (4.0, 44.0) | 9.0 (5.0, 19.0) |
| Days (min, max) | (1, 115) | (4, 68) |
| Insomnia, *n*^a^ | 46 | 18 |
| Median days (P10, P90) | 27.0 (5.0, 72.0) | 10.5 (2.0, 311.0) |
| Days (min, max) | (2, 243) | (1, 336) |
| Dry mouth, *n*^a^ | 44 | 16 |
| Median days (P10, P90) | 31.0 (3.0, 195.0) | 91.0 (9.0, 371.0) |
| Days (min, max) | (1, 373) | (2, 381) |
| Fatigue, *n*^a^ | 47 | 17 |
| Median days (P10, P90) | 24.0 (6.0, 203.0) | 33.0 (7.0, 178.0) |
| Days (min, max) | (2, 398) | (4, 215) |
| Somnolence, *n*^a^ | 85 | 20 |
| Median days (P10, P90) | 17.0 (2.0, 153.0) | 37.0 (15.5, 192.0) |
| Days (min, max) | (1, 357) | (3, 359) |

^a^*n* refers to number of patients reporting event, not number of events

Adverse events were coded by preferred terms and system organ class using the *Medical Dictionary for Regulatory Activities*, version 19.0

For those patients who emerge multiple times for the same adverse event, the adverse event with the longest duration during treatment period was chosen

*BUP* buprenorphine, *max* maximum, *min* minimum, *P10* 10th percentile, *P90* 90th percentile, *SAM* samidorphan

**Table S2.** Adverse events leading to discontinuation

| **Patients with event, n (%)** | **No prior exposure to BUP/SAM**  **(*n* = 929)** | **Prior exposure to BUP/SAM**  **(*n* = 556)** | **All patients**  **(*N* = 1485)** |
| --- | --- | --- | --- |
| Any AE leading to study discontinuation | 122 (13.1) | 32 (5.8) | 154 (10.4) |
| Nausea | 41 (4.4) | 7 (1.3) | 48 (3.2) |
| Dizziness | 18 (1.9) | 1 (0.2) | 19 (1.3) |
| Vomiting | 18 (1.9) | 1 (0.2) | 19 (1.3) |
| Somnolence | 9 (1.0) | 1 (0.2) | 10 (0.7) |
| Fatigue | 6 (0.6) | 1 (0.2) | 7 (0.5) |
| Constipation | 6 (0.6) | 0 | 6 (0.4) |
| Headache | 4 (0.4) | 2 (0.4) | 6 (0.4) |
| Anxiety | 3 (0.3) | 1 (0.2) | 4 (0.3) |
| Depression | 4 (0.4) | 0 | 4 (0.3) |
| Palpitations | 3 (0.3) | 1 (0.2) | 4 (0.3) |
| Suicidal ideation | 4 (0.4) | 0 | 4 (0.3) |
| Tremor | 2 (0.2) | 2 (0.4) | 4 (0.3) |
| Hyperhidrosis | 3 (0.3) | 0 | 3 (0.2) |
| Malaise | 3 (0.3) | 1 (0.2) | 3 (0.2) |

AEs were coded by preferred terms and system organ class using the *Medical Dictionary for Regulatory Activities*, version 19.0

*AE* adverse event, *BUP* buprenorphine, *SAM* samidorphan

**Table S3.** Serious adverse events reported during the treatment period in ≥1 patient in any treatment group

| **Patients with event, *n* (%)** | **No prior exposure to BUP/SAM**  **(*n* = 929)** | **Prior exposure  to BUP/SAM**  **(*n* = 556)** | **All patients**  **(*N* = 1485)** |
| --- | --- | --- | --- |
| Any SAE | 33 (3.6) | 14 (2.5) | 47 (3.2) |
| Specific SAEs (≥1 in any treatment group) | | | |
| Depression | 2 (0.2) | 1 (0.2) | 3 (0.2) |
| Suicidal ideation | 3 (0.3) | 0 | 3 (0.2) |
| Colitis | 2 (0.2) | 0 | 2 (0.1) |
| Myocardial infarction | 1 (0.1) | 1 (0.2) | 2 (0.1) |
| Pneumonia | 2 (0.2) | 0 | 2 (0.1) |
| Sepsis | 2 (0.2) | 0 | 2 (0.1) |
| Uterine leiomyoma | 2 (0.2) | 0 | 2 (0.1) |
| Abortion missed | 1 (0.1) | 0 | 1 (0.1) |
| Abortion spontaneous | 0 | 1 (0.2) | 1 (0.1) |
| Alcohol poisoning | 1 (0.1) | 0 | 1 (0.1) |
| Anemia | 1 (0.1) | 0 | 1 (0.1) |
| Ankle fracture | 0 | 1 (0.2) | 1 (0.1) |
| Appendicitis | 1 (0.1) | 0 | 1 (0.1) |
| Atrial fibrillation | 1 (0.1) | 0 | 1 (0.1) |
| Breast cancer | 1 (0.1) | 0 | 1 (0.1) |
| Cardiac failure congestive | 0 | 1 (0.2) | 1 (0.1) |
| Cellulitis | 0 | 1 (0.2) | 1 (0.1) |
| Cerebral hemorrhage | 1 (0.1) | 0 | 1 (0.1) |
| Chest pain | 0 | 1 (0.2) | 1 (0.1) |
| Cholelithiasis | 1 (0.1) | 0 | 1 (0.1) |
| Conversion disorder | 0 | 1 (0.2) | 1 (0.1) |
| Diabetic ketoacidosis | 1 (0.1) | 0 | 1 (0.1) |
| Duodenal ulcer | 1 (0.1) | 0 | 1 (0.1) |
| Ectopic pregnancy | 1 (0.1) | 0 | 1 (0.1) |
| Endometriosis | 1 (0.1) | 0 | 1 (0.1) |
| Esophageal adenocarcinoma | 0 | 1 (0.2) | 1 (0.1) |
| Gastric ulcer hemorrhage | 1 (0.1) | 0 | 1 (0.1) |
| Ligament sprain | 0 | 1 (0.2) | 1 (0.1) |
| Lumbar spinal stenosis | 1 (0.1) | 0 | 1 (0.1) |
| Major depression | 1 (0.1) | 0 | 1 (0.1) |
| Meningitis viral | 1 (0.1) | 0 | 1 (0.1) |
| Menometrorrhagia | 1 (0.1) | 0 | 1 (0.1) |
| Menorrhagia | 1 (0.1) | 0 | 1 (0.1) |
| Mental status changes | 0 | 1 (0.2) | 1 (0.1) |
| Osteomyelitis | 1 (0.1) | 0 | 1 (0.1) |
| Otitis media | 1 (0.1) | 0 | 1 (0.1) |
| Pancreatitis | 0 | 1 (0.2) | 1 (0.1) |
| Phlebitis | 1 (0.1) | 0 | 1 (0.1) |
| Pulmonary edema | 1 (0.1) | 0 | 1 (0.1) |
| Pyelonephritis | 0 | 1 (0.2) | 1 (0.1) |
| Pyrexia | 0 | 1 (0.2) | 1 (0.1) |
| Respiratory arrest | 1 (0.1) | 0 | 1 (0.1) |
| Seminoma | 0 | 1 (0.2) | 1 (0.1) |
| Sinus tachycardia | 1 (0.1) | 0 | 1 (0.1) |
| Small intestinal obstruction | 1 (0.1) | 0 | 1 (0.1) |
| Uterine prolapse | 0 | 1 (0.2) | 1 (0.1) |
| Vaginal prolapse | 0 | 1 (0.2) | 1 (0.1) |

Adverse events were coded by preferred terms and system organ class using the *Medical Dictionary for Regulatory Activities,* version 19.0

*BUP* buprenorphine, *SAE* serious adverse event, *SAM* samidorphan

**Table S4.** Suicide-related events based on Columbia Suicide Severity Rating Scale (C-SSRS) during treatment period

| **Treatment-emergent suicide-related events** | **All patients (*N* = 1485) *n*/*m*^a^ (%)** |
| --- | --- |
| Increase from baseline in suicidal ideation | 100/1475 (6.8) |
| Emergence of serious suicidal ideation | 2/1475 (0.1) |
| Emergence of serious suicidal ideation in patients with no suicidal ideation at baseline | 1/1404 (0.1) |
| Decrease from baseline in suicidal ideation | 58/71 (81.7) |
| Emergence of suicidal behavior in patients with no suicidal behavior at baseline | 1/1475 (0.1) |

^a^*n* is defined as the number of patients with a nonmissing baseline and postbaseline C-SSRS result, and *m* is defined as the number of patients with a nonmissing baseline and at least one postbaseline C-SSRS assessment during the treatment period meeting the suicide-related denominator criteria for each category

**Table S5.** Clinical Opiate Withdrawal Scale (COWS) score by time interval during postdiscontinuation safety period

| **Parameter** | **No prior exposure to BUP/SAM (*n* = 527)** | **Prior exposure to BUP/SAM (*n* = 343)** | **All patients (*N* = 870)** |
| --- | --- | --- | --- |
| Baseline | | | |
| Patients, n | 527 | 343 | 870 |
| COWS score |  |  |  |
| Mean (SD) | 0.5 (0.98) | 0.5 (0.98) | 0.5 (0.98) |
| Median (min, max) | 0 (0, 8) | 0 (0, 7) | 0 (0, 8) |
| Days between last dose and assessment date | | | |
| Mean (SD) | 0.9 (0.33) | 1.0 (0.51) | 1.0 (0.41) |
| 1–5 days post last dose | | | |
| Patients, n | 511 | 337 | 848 |
| COWS score |  |  |  |
| Mean (SD) | 0.7 (1.41) | 0.6 (1.26) | 0.7 (1.36) |
| Median (min, max) | 0 (0, 12) | 0 (0, 9) | 0 (0, 12) |
| Change from baseline COWS score | | | |
| Mean (SD) | 0.2 (1.30) | 0.1 (1.19) | 0.2 (1.26) |
| Median (min, max) | 0 (–4, 12) | 0 (–5, 9) | 0 (–5, 12) |
| Days between last dose and time interval assessment date | | | |
| Mean (SD) | 2.2 (0.68) | 2.2 (0.69) | 2.2 (0.69) |
| 6–10 days post last dose | | | |
| Patients, n | 503 | 321 | 824 |
| COWS score |  |  |  |
| Mean (SD) | 0.9 (1.56) | 0.8 (1.65) | 0.8 (1.59) |
| Median (min, max) | 0 (0, 9) | 0 (0, 14) | 0 (0, 14) |
| Change from baseline COWS score | | | |
| Mean (SD) | 0.3 (1.50) | 0.3 (1.71) | 0.3 (1.58) |
| Median (min, max) | 0 (–7, 8) | 0 (–5, 14) | 0 (–7, 14) |
| Days between last dose and time interval assessment date | | | |
| Mean (SD) | 8.5 (0.78) | 8.5 (0.78) | 8.5 (0.78) |
| 11–16 days post last dose | | | |
| Patients, n | 453 | 284 | 737 |
| COWS score |  |  |  |
| Mean (SD) | 0.9 (1.98) | 0.9 (1.86) | 0.9 (1.94) |
| Median (min, max) | 0 (0, 19) | 0 (0, 15) | 0 (0, 19) |
| Change from baseline COWS score | | | |
| Mean (SD) | 0.4 (1.90) | 0.3 (1.81) | 0.4 (1.86) |
| Median (min, max) | 0 (–7, 16) | 0 (–5, 15) | 0 (–7, 16) |
| Days between last dose and time interval assessment date | | | |
| Mean (SD) | 15.0 (0.92) | 15.1 (0.97) | 15.1 (0.94) |
| 17–23 days post last dose | | | |
| Patients, n | 87 | 72 | 159 |
| COWS score |  |  |  |
| Mean (SD) | 0.7 (1.24) | 0.6 (0.87) | 0.7 (1.09) |
| Median (min, max) | 0 (0, 5) | 0 (0, 4) | 0 (0, 5) |
| Change from baseline COWS score | | | |
| Mean (SD) | 0.1 (1.26) | 0.0 (1.12) | 0.1 (1.19) |
| Median (min, max) | 0 (–3, 5) | 0 (–5, 2) | 0 (–5, 5) |
| Days between last dose and time interval assessment date | | | |
| Mean (SD) | 18.9 (2.27) | 18.8 (2.15) | 18.8 (2.21) |
| ≥24 days post last dose | | | |
| Patients, n | 487 | 326 | 813 |
| COWS score |  |  |  |
| Mean (SD) | 0.7 (1.67) | 0.6 (1.49) | 0.6 (1.60) |
| Median (min, max) | 0 (0, 16) | 0 (0, 13) | 0 (0, 16) |
| Change from baseline COWS score | | | |
| Mean (SD) | 0.2 (1.63) | 0.1 (1.56) | 0.1 (1.60) |
| Median (min, max) | 0 (–8, 14) | 0 (–5, 13) | 0 (–8, 14) |
| Days between last dose and time interval assessment date | | | |
| Mean (SD) | 30.6 (7.20) | 30.5 (5.20) | 30.5 (6.47) |

Patients included only those with exposure ≥4 weeks

^a^Baseline is defined as the time of COWS first assessment after end of treatment

^b^Days between last dose and assessment date are calculated as date of assessment – date of last dose of study drug

*BUP* buprenorphine, *max* maximum, *min* minimum, *SAM* samidorphan, *SD* standard deviation

**Table S6.** Patients with potentially clinically significant values in weight at and after baseline assessment during treatment period

| **Baseline BMI category**  **PCS criteria** | **No prior exposure to BUP/SAM (*n* = 929) *n*/*m*^a^ (%)** | **Prior exposure  to BUP/SAM (*n* = 556) *n*/*m*^a^ (%)** | **All patients (*N* = 1485) *n*/*m*^a^ (%)** |  |
| --- | --- | --- | --- | --- |
| All patients^b^ |  |  |  |  |
| ≥7% increase from baseline | 105/924 (11.4) | 48/552 (8.7) | 153/1476 (10.4) |  |
| ≥7% decrease from baseline | 91/924 (9.8) | 55/552 (10.0) | 146/1476 (9.9) |  |
| Underweight (<18.5 kg/m^2^) |  |  |  |  |
| ≥7% increase from baseline | 2/3 (66.7) | 0/2 | 2/5 (40.0) |  |
| ≥7% decrease from baseline | 0/3 | 0/2 | 0/5 |  |
| Normal (≥18.5 to <25 kg/m^2^) |  |  |  |  |
| ≥7% increase from baseline | 29/223 (13.0) | 18/130 (13.8) | 47/353 (13.3) |  |
| ≥7% decrease from baseline | 14/223 (6.3) | 10/130 (7.7) | 24/353 (6.8) |  |
| Overweight (≥25 to <30 kg/m^2^) |  |  |  |  |
| ≥7% increase from baseline | 40/284 (14.1) | 15/171 (8.8) | 55/455 (12.1) |  |
| ≥7% decrease from baseline | 31/284 (10.9) | 17/171 (9.9) | 48/455 (10.5) |  |
| Obese (≥30 kg/m^2^) |  |  |  |  |
| ≥7% increase from baseline | 34/414 (8.2) | 15/249 (6.0) | 49/663 (7.4) |  |
| ≥7% decrease from baseline | 46/414 (11.1) | 28/249 (11.2) | 74/663 (11.2) |  |

^a^*n* is the number of patients who met the PCS criteria in the baseline BMI category. *m* is the number of patients in the baseline BMI category with baseline and at least one postbaseline assessment

^b^For body weight at last visit, see Table S8

*BMI* body mass index, *BUP* buprenorphine, *PCS* potentially clinically significant, *SAM* samidorphan

**Table S7.** Patients with potentially clinically significant values in cholesterol at last visit

| **Parameter**  **PCS criteria** | **No prior exposure  to BUP/SAM (*n* = 929) *n*/*m*^a^ (%)** | **Prior exposure  to BUP/SAM (*n* = 556) *n*/*m*^a^ (%)** | **All patients (*N* = 1485) *n*/*m*^a^ (%)** |
| --- | --- | --- | --- |
| Cholesterol, total  >300 mg/dl | 10/897 (1.1) | 4/540 (0.7) | 14/1437 (1.0) |
| HDL cholesterol  ≤30 mg/dl | 10/892 (1.1) | 9/543 (1.7) | 19/1435 (1.3) |
| LDL cholesterol  ≥160 mg/dl | 46/747 (6.2) | 30/452 (6.6) | 76/1199 (6.3) |
| Triglycerides |  |  |  |
| >500 mg/dl | 6/899 (0.7) | 6/546 (1.1) | 12/1445 (0.8) |

^a^*n* is the number of patients who met the PCS criteria in the baseline BMI category. *m* is the number of patients in the baseline BMI category with baseline and at least one postbaseline assessment

*BUP* buprenorphine, *HDL* high-density lipoprotein, *LDL* low-density lipoprotein, *PCS* potentially clinically significant, SAM, samidorphan

**Table S8.** Patients with potentially clinically significant values in vital signs and body weight at last visit

| **Parameter/PCS criteria** | **No prior exposure  to BUP/SAM (*n* = 929) *n*/*m*^a^ (%)** | **Prior exposure  to BUP/SAM (*n* = 556) *n*/*m*^a^ (%)** | **All patients (*N* = 1485) *n*/*m*^a^ (%)** |
| --- | --- | --- | --- |
| Supine systolic blood pressure |  |  |  |
| Low: ≤90 mmHg and decrease ≥20 mmHg | 0/922 | 1/549 (0.2) | 1/1471 (0.1) |
| High: ≥140 mmHg and increase ≥20 mmHg | 28/838 (3.3) | 22/507 (4.3) | 50/1345 (3.7) |
| Supine diastolic blood pressure |  |  |  |
| Low: ≤50 mmHg and decrease ≥10 mmHg | 2/923 (0.2) | 0/551 | 2/1474 (0.1) |
| High: ≥90 mmHg and increase ≥10 mmHg | 30/835 (3.6) | 20/508 (3.9) | 50/1343 (3.7) |
| Heart rate |  |  |  |
| Low: ≤50 bpm and decrease ≥15 bpm | 1/911 (0.1) | 0/546 | 1/1457 (0.1) |
| High: ≥100 bpm and increase ≥15 bpm | 7/918 (0.8) | 4/552 (0.7) | 11/1470 (0.7) |
| Temperature |  |  |  |
| Hypothermia: ≤35.0°C | 0/924 | 1/552 (0.2) | 1/1476 (0.1) |
| Hyperthermia: ≥38.1°C | 1/924 (0.1) | 0/552 | 1/1476 (0.1) |
| Respiratory rate |  |  |  |
| Low: <12 breaths per minute | 1/924 (0.1) | 1/552 (0.2) | 2/1476 (0.1) |
| High: >25 breaths per minute | 0/924 | 1/552 (0.2) | 1/1476 (0.1) |
| Body weight^b^ |  |  |  |
| ≥7% increase from baseline | 85/924 (9.2) | 38/552 (6.9) | 123/1476 (8.3) |
| ≥7% decrease from baseline | 58/924 (6.3) | 32/552 (5.8) | 90/1476 (6.1) |

^a^*n* is the number of patients who met the PCS criteria in the baseline body mass index category. *m* is the number of patients in the baseline body mass index category with baseline and at least one postbaseline assessment

^b^For body weight increases during the treatment period (i.e., not just at last visit) see Table S6

*BUP* buprenorphine, *PCS* potentially clinically significant, *SAM* samidorphan

**Fig. S1.** Patient disposition.


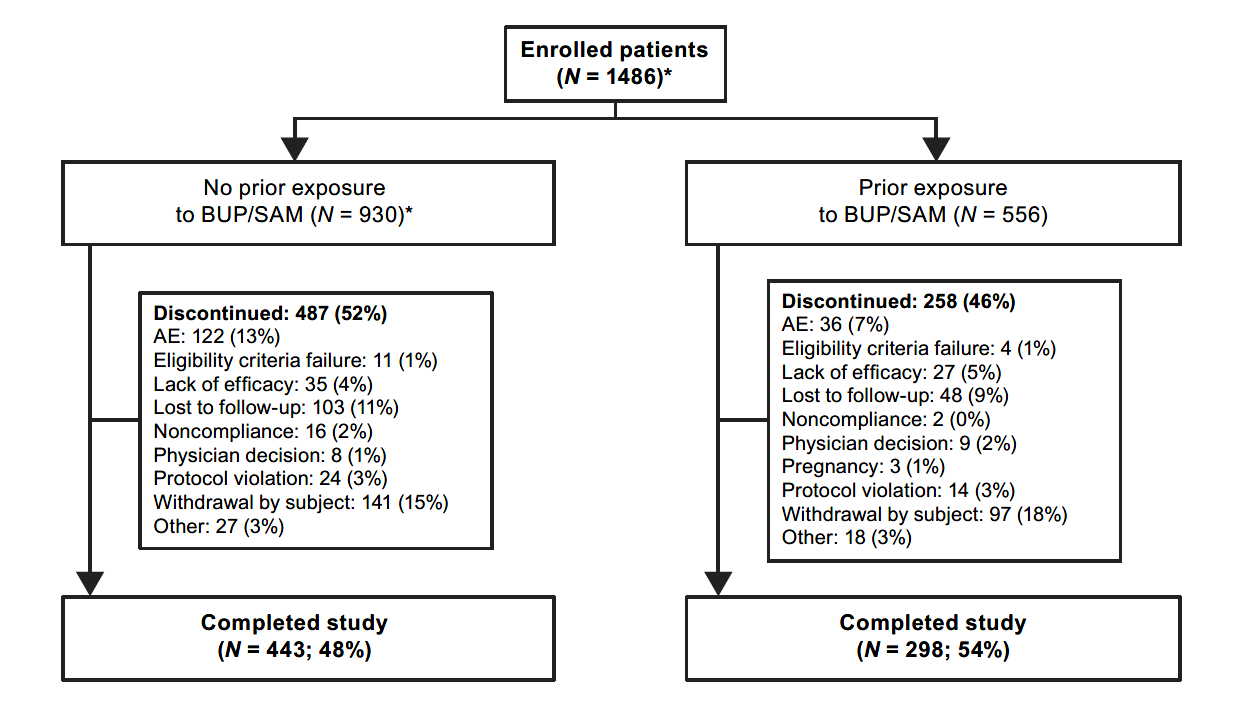


^a^One patient enrolled but did not enter the treatment phase of study.

*AE* adverse event, *BUP* buprenorphine, *SAM* samidorphan.

**Fig. S2.** Time to treatment discontinuation^a^.


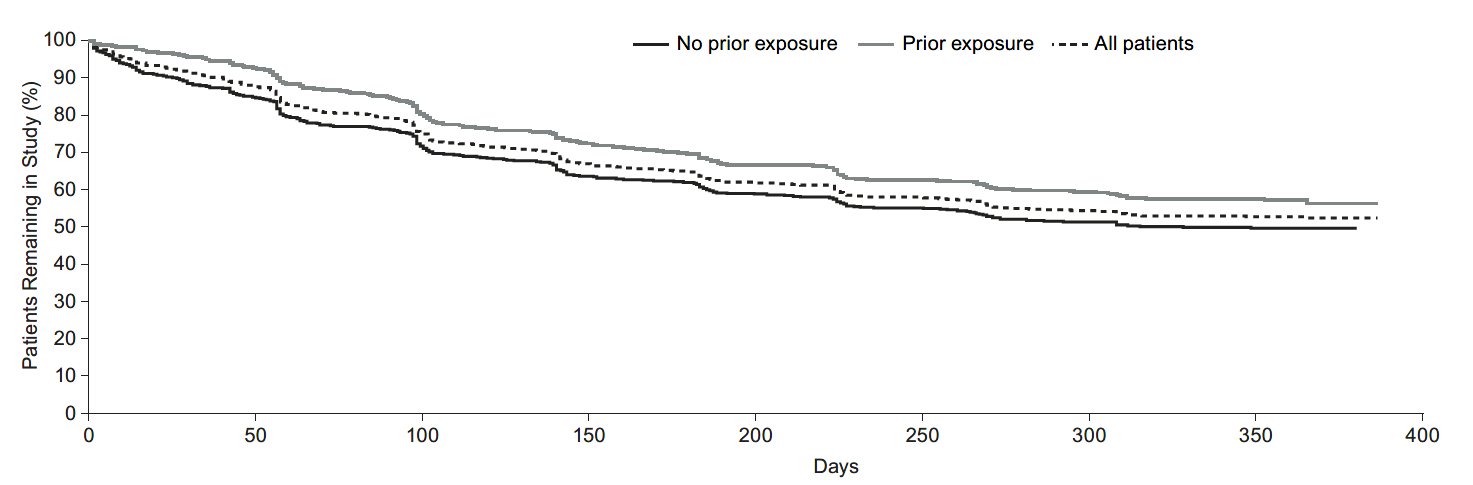


^a^Time to discontinuation estimated using Kaplan–Meier methods.

1. [↑](#footnote-ref-1)
2. [↑](#footnote-ref-2)
