# Supplementary figures and images for: Results from a long-term open-label extension study of adjunctive buprenorphine/samidorphan combination in patients with major depressive disorder
Source: Neuropsychopharmacology. 2019 Jun 29;44(13):2268–76. doi: 10.1038/s41386-019-0451-3 (PMC6897901; doi:10.1038/s41386-019-0451-3)

Supplemental Figure 1

Patient Disposition

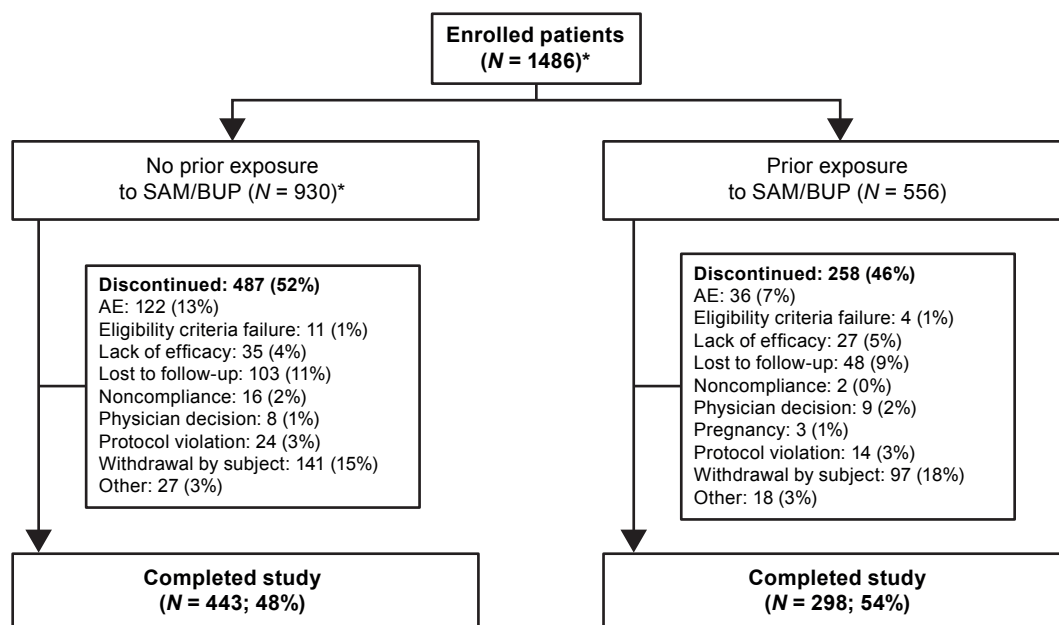

Supplement: Supplementary file 2 — Supplemental Figure 1 [file 41386_2019_451_MOESM2_ESM.pdf]

Supplemental Figure 2  
Patient Retention Over Time

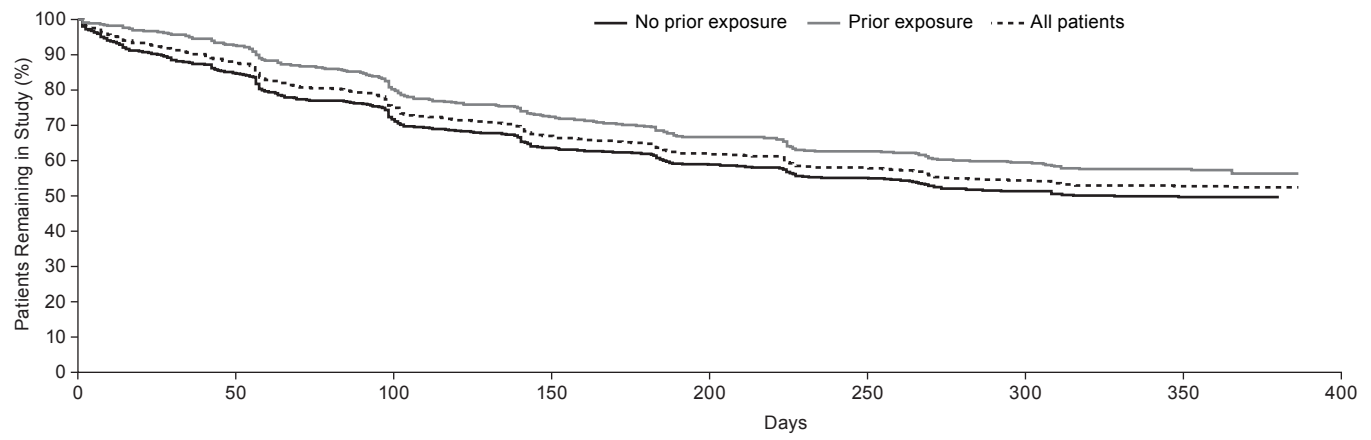

Supplement: Supplementary file 3 — Supplemental Figure 2 [file 41386_2019_451_MOESM3_ESM.pdf]
